# Supplementary material for: De-novo non-convulsive status epilepticus in adult medical inpatients without known epilepsy: Analysis of mortality related factors and literature review
Source: PLoS One. 2021 Oct 15;16(10):e0258602. doi: 10.1371/journal.pone.0258602 (PMC8519439; doi:10.1371/journal.pone.0258602)
Supplement: S1 Table — NP: not performed, NR: not reported, D: day, W: week, Min: minutes, LEV: levetiracetam, LCM: lacosamide, PHT: Phenytoin, MPHT: mephenytoin, VPA: Valproic acid, BZD: benzodiazepines, PB: phenobarbital, DXM: dexamethasone, DZP: diazepam, THP: thiopental, PPF: propofol, PPH: phospho-phenytoin, MDZ: midazolam, LMG: lamotrigine, RPD: risperidone, HPD: Haloperidol, MTP: methylprednisolone, PDN: prednisone, CyC: cyclophosphamide, IVIG: immunoglobulins, RTX: rituximab, CLZ: clonazepam, CLP: chlorpromazine, QCN: quinacrine, PLPH: plasmapheresis, ZND: zonisamide, FBT: felbamate, PTB: pentobarbital, GBP: gabapentin, DPH: diphenylhydantoin, STE: steroids, NLX: naloxone, (ISN + RIF + PYR + ETB): isoniazid, rifampicin, pyrazinamide and ethambutol, SIRPID: stimulus-induced rhythmic, periodic, or ictal discharges, PRES: posterior reversible encephalopathy syndrome, UTI: urinary tract infection, ACS: acute coronary syndrome. *Global data from Canas N. et al: 6 patients admitted in ICU, 9 patients died. **Global data from Labar D. et al: 3 patients died and 2 presented a complete recovery. (PDF) [file pone.0258602.s002.pdf]

Supplementary Table S1. Cases included in the literature review by main etiology.

**Etiology**		**Study**	**N**	**Year**	**Country**	**Age**	**Diagnosis**	**Treatment**	**ICU**	**Evolution**
Antibiotics	Cephalosporins	Cunningham JM. *et al* [1]	1	2020	USA	72	<1d	LOR, LEV<br>Cefepime discontinued	No	Complete recovery in 1d
		Tchapyjnikov D. *et al* [2]	3	2019	USA	59	<1d (n=3)	Cefepime discontinued (n=3)<br>LOR (n=2)<br>LEV (n=1)	NR (n=3)	Complete recovery in 1d (n=2)<br>Complete recovery in 5d (n=1)
		Skaistys A. *et al* [3]	1	2019	Germany	83	NR	LEV, LCM<br>Cefepime discontinued	Yes	Died in 16d
		Hagiya H. *et al* [4]	1	2017	Japan	56	3d	Ceftriaxone discontinued	Yes	Complete recovery in 5d
		Collins RD. et *al* [5]	1	2016	USA	64	<1d	PHT, MDZ, PPF, LEV, LCM<br>Ceftazidime discontinued	Yes	Complete recovery in 15d
		Fernández-Torre JL. *et al* [6]	3	2012	Spain	52	<1d	PHT, CLZ	NR	Complete recovery in 1d
		Balderia PG. *et al* [7]	1	2015	USA	49	<1d	LOR, LEV<br>Cefepime discontinued	NR	Complete recovery in 5d
		Kim A. *et al* [8]	1	2013	Korea	71	<1d	LOR, LEV, VPA, PHT, CLZ	NR	Complete recovery in 3d
		Chang YM. *et al* [9]	1	2013	Taiwan	86	2d	LOR, PHT<br>Cefepime discontinued	No	Complete recovery in 1w
		Kim KB. *et al* [10]	1	2012	Korea	69	1d	Ceftriaxone discontinued	NR	Complete recovery in 2d
		Sethi NK. *et al* [11]	1	2010	USA	72	<1d	BZD	NR	Complete recovery in 2d

		Spriet I. *et al* [12]	2	2007	Belgium	57	<1d	VPA (n=2), LEV, PHT, PTB (n=1) Cefepime discontinued (n=2)	Yes (n=1) NR (n=1)	Died in 33d (n=1) Died in 11d (n=1)
		Iftikhar S. *et al* [13]	1	2007	Saudi Arabia	48	<1d	DZP, PHT	NR	Complete recovery in 2d
		Vannaprasaht S. *et al* [14]	1	2006	Thailand	70	2d	PHT, DZP, ceftazidime discontinued	Yes	Complete recovery in 14d
		Maganti R. *et. al* [15]	1	2005	USA	79	1d	LOR, VPA	NR	Complete recovery in 5d
		Primavera A. *et al* [16]	2	2004	Italy	73	1d (n=2)	LOR, ceftazidime discontinued (n=1) DZP, hemodialysis, cefepime discontinued (n=1)	NR (n=2)	Complete recovery in 2d (n=1) Complete recovery in 1w (n=1)
		Abanades S. *et al* [17]	1	2004	Spain	66	1d	CLZ, VPA, Cefepime discontinued	Yes	Complete recovery in 2d
		Plensa E. *et al* [18]	1	2004	Spain	65	<1d	PHT, cefepime discontinued	Yes	Complete recovery in 1m
		Ferrara N. *et al* [19]	1	2003	Italy	82	<1d	cefepime discontinued, hemodialysis	NR	Complete recovery in 1d
		Chow KM. *et al* [20]	4	2001	China	53	2d (n=4)	PHT (n=4) DZP, LOR (n=2) PB (n=1) Cefepime discontinued (n=1)	NR (n=4)	Complete recovery in 1-2d (n=2) Partial recovery (n=2)
		Chatellier D. *et al* [21]	2	2001	France	69	NR (n=2)	CLZ, Hemodialysis	Yes (n=2)	Complete recovery (n=1) Died (n=1)

		Martínez-Rodriguez JE. *et al* [22]	2	2001	Spain	81	NR (n=2)	PHT (n=1)<br>VPA (n=2)<br>Ceftriaxone discontinued (n=2)	NR (n=2)	Complete recovery in 5d (n=2)
		Saurina A. *et al* [23]	2	2000	Spain	71	NR	CLZ, cefepime discontinued (n=2)<br>VPA, PHT (n=1)	NR	Complete recovery in 3d<br>Partial recovery in 4d
		Dixit S. *et al* [24]	1	2000	USA	44	2d	LOR, VPA	NR	Complete recovery in 1w
		Klion AD. *et al* [25]	1	1994	USA	77	12d	PHT, DZP Ceftazidime discontinued	NR	Complete recovery in 34d
	Penicillins	Rissardo JP. *et al* [26]	1	2019	Brazil	23	<1d	MDZ, PHT, PPF<br>Penicillin discontinued	Yes	NR
		Gürses C. *et al* [27]	1	2007	Turkey	71	NR	PHT<br>Penicillin discontinued	NR	Partial recovery
		Kojan S. *et al* [28]	1	2000	USA	55	<1d	PPH, MTP<br>PDN<br>Penicillin discontinued	NR	Partial recovery in 7d
		Zifko U. *et al* [29]	1	1994	Austria	40	1d	NR	NR	Complete recovery in 2w
Chemotherapy	Cisplatinum	Holman L. *et al* [30]	1	2015	USA/China	54	<1d	LOR, LEV,<br>Discontinued cisplatin	NR	Complete recovery in 3d
	Cetuximab	Palma JA. *et al* [31]	1	2011	Spain	40	<1d	CZP, VPA, PPF, LEV	Yes	Complete recovery in 7d
	Ifosfamide	Taupin D. *et al* [32]	1	2013	USA	60	3	Methylene blue, thiamine, LEV, MDZ. Discontinued ifosfamide	No	Complete recovery in 1w

		Kilickap S. *et al* [33]	2	2006	Turkey	54	<1d	DZP + LEV ifosfamide discontinued (n=2)	NR (n=2)	Complete recovery in 3d
		Primavera A. *et al* [16]	2	2002	Italy	27 / 57	2d (n=2)	DZP, ifosfamide discontinued (n=2)	NR (n=2)	Complete recovery in 2d (n=1)<br>Partial recovery in 1d (n=1)
		Bhardwaj A. *et al* [34]	1	1995	USA	71	<1d	PHT and ifosfamide discontinued	NR	Complete recovery in 3d
		Wengs W.J. *et al* [35]	1	1993	USA	57	<1d	DZP	No	Complete recovery in 2d
Other Drugs	Antidepressant	Yoshino A. *et al* [36]	3	1997	Japan	48	NR (n=3)	DZP (n=2)<br>Antidepressant (n=1)	NR (n=3)	Complete recovery in 1w (n=2)<br>Complete recovery in 2w (n=1)
	Baclofen	Zak R. *et al* [37]	1	1994	USA	50	<1d	DZP, PHT	NR	Complete recovery in <1d
	Chloroquine	Benbadis SR. *et al* [38]	1	1996	Switzerland	68	4d	CLZ, CBZ	NR	Complete recovery in <1d
	Fentanyl	Pogliani D. *et al* [39]	2	2016	Italy	79	<1d (n=1)<br>3d (n=1)	LOR (n=1)<br>DZP - DXM (n=1)	NR (n=2)	Complete recovery in 5d (n=1)<br>Died in 9d (n=1)
	Levofloxacin	Fernández-Torre JL. *et al* [6]	1	2012	Spain	73	1d	PHT, DZP	NR	Complete recovery I 1d

	Lithium	Roccatagliata L. *et al* [40]	1	2002	Italy	84	2d	DZP	NR	Complete recovery in <1d
	Methotrexate	Patterson DM. *et al* [41]	1	2011	UK	58	NR	DZP	NR	Complete recovery but recurrence by the 5 month
	Morphine	Bertran F. *et al* [42]	1	1999	France	77	<1d	CLZ, NLX	NR	Complete recovery in 6d
	Theophylline	Krieger AC. *et al* [43]	1	1999	USA	53	<1d	MDZ, PHT, PB	Yes	Complete recovery in 2w
		Hilkens PHE. *et al* [44]	1	1995	The Netherlands	84	1d	CBZ, CLZ	NR	Complete recovery in 5d
Drug-Related Syndromes	Neuroleptic Malignant Syndrome	Mogi T. *et al* [45]	1	2017	Japan	44	NR	MDZ	NR	Complete recovery in 22d
		Yoshino A. *et al* [46]	1	2000	Japan	62	<1d	DZP	NR	Complete recovery in <1d
	BZD withdrawal	Olnes MJ. *et al* [47]	1	2003	USA	64	Several days	LOR	NR	Complete recovery in min
		Fernández-Torre JL. *et al* [48]	1	2001	Spain	79	3d	DZP, PHT	NR	Complete recovery in <1d
		Kanemoto K. *et al* [49]	1	1999	Japan	78	1w	DZP	No	Complete recovery
		Primavera A. *et al* [50]	1	1995	Italy	76	2d	DZP, PB	NR	Complete recovery in 1m
Contrasts	Iothalmate meglumine	Vickrey BG. *et al* [51]	1	1989	USA	64	<1d	PHT	NR	Complete recovery in <1d

	Metrizamide	Amer-Ferrer G. *et al* [52]	1	1988	Spain	48	<1d	CLZ	NR	Complete recovery in 3w
		Levin R. *et al* [53]	1	1985	USA	68	2d	DZP, PHT	NR	Complete recovery in 1d
		Pritchard PB. *et al* [54]	1	1984	USA	59	<1d	DPZ, PHT	NR	Complete recovery in 2d
Toxics	Carbon monoxide	Brown K.L. *et al* [55]	1	2007	USA	76	<1d	MDZ	Yes	Died in 28d
	Cocaine	Uthman B. *et al* [56]	1	2007	USA	43	9d	LOR, VPA	NR	Died in 7w
	Metaldehyde	Fernández-Torre JL. *et al* [57]	1	2018	Spain	68	<1d	PPF, LEV, PHT	Yes	Complete recovery in 15d
	Nickel	Denays R. *et al* [58]	1	2005	Belgium	43	<1d	DZP, PHT	NR	Complete recovery in 4d
	SESA syndrome	Kaplan PW. *et al* [59]	2	2018	USA	52	2d (n=2)	LEV (n=1)<br>LOR, PPHT, PHT, LCM, DZP, Thiamine, folate (n=1)	Yes (n=1)<br>NR (n=1)	Partial recovery in 2w (n=1)<br>Complete recovery in 8d
		Fernández-Torre JL. *et al* [6]	2	2012	Spain	58	1d	PHT, VPA	NR	Complete recovery in 1-2d
		Fernández-Torre JL. *et al* [60]	2	2014	Spain	57	<1d (n=2)	PHT, LEV (n=2)<br>PPF (n=1)	Yes (n=2)	Recovery in 1-2d
Infections	AIDS dementia	Wong MC. *et al* [61]	1	1992	USA	NR	NR	DZP	NR	Complete recovery in <1d
	Creutzfeldt-Jakob	Sharma DK. *et al* [62]	1	2017	Australia	68	NR	CLZ, LEV, PHT, MTP, IVIG	NR	Died in 3m

		Albanese M. *et al* [63]	1	2014	Italy	66	3m	DZP, VPA	NR	Complete recovery in 6m
		Aiguabella M. *et al* [64]	1	2010	Spain	44	<1d	CLO, PHE, LEV, MDZ	Yes	Died in 25d
		Espinosa PS. *et al* [65]	1	2010	USA	64	1w	PPF, PHT, PB, VPA, TOP, LEV	Yes	Died in 3w
		Rossetti A. *et al* [66]	1	2007	Switzerland	74	3w	PHT, PPF, CLZ	Yes	Died in 2w
		Cohen D. *et al* [67]	1	2004	USA	26	2m	TOP, PB, OXC, LEV, PPH, VPA, MDZ, CLP, QCN	Yes	Died in 76d
		Shapiro JM. *et al* [68]	1	2004	USA	71	1m	LOR, MDZ, PHT, LEV	Yes	Died in 1m
	Cryptococcosis	Cury RF. *et al* [69]	1	2003	Brazil	37	2d	PHT, DZP, MDZ, PHT	Yes	Died in 41d
	Dengue	Assenza F. *et al* [70]	1	2016	Italy	79	Some days	DZM, LEV, VPA, PB	Yes	Died in 3m
	Fever	Riggio S. *et al* [71]	1	2006	USA	83	<1d	PHT	NR	Recovery
	Herpes encephalitis	Lee JH. *et al* [72]	1	2012	Korea	19	Several days	Acyclovir, PHT, LOR	NR	Recovery
	Neurosyphilis	Primavera A. *et al* [73]	1	1998	Italy	44	<1d	Penicilin G, PB	Yes	Recovery
	Postinfectious encephalitis	Wusthoff CJ. *et al* [74]	1	2009	USA	29	<1d	LOR, PHT, VPA, MDZ PB, TOP, LEV, PTB, Ketogenic diet	Yes	Partial recovery in 26d
	Tuberculous meningitis	Arman F. *et al* [75]	1	2011	Italy	66	6m	DZP + PHE + MDZ + (ISN + RIF + PYR + ETB)	NR	Partial recovery
	Urinay tract infection	Shea Y. *et al* [76]	1	2016	China	86	11d	PHT, DZM, VPA	NR	Partial recovery
	CNS infection	Fernández-Torre JL. *et al* [6]	3	2012	Spain	60	1d	PHT, VPA, CLZ, PPF, THP	NR	Partial recovery (n=19)<br>Died (n=2)

	Sepsis	Lin Y. *et al* [77]	1	2015	Taiwan	68	4d	LEV, ZND, CLZ, STE	NR	Partial recovery in 3m
		Fernández-Torre JL. *et al* [6]	1	2012	Spain	75	1d	PHT, DZP	NR	Died
		Korn-Lubetzki I. *et al* [78]	1	2007	Israel	81	5d	DZP, PHT, VPA	No	Died
	Viral encephalitis	Arif H. *et al* [79]	1	2008	USA	63	<1d	LOR, PPF, VPA, LEV, TOP, OXC, PTB	Yes	Partial recovery in 2m
Autoimmunity	Anti-NMDA Receptor Encephalitis	Shi Y. *et al* [80]	1	2017	USA	20	1w	PLPH, IVIG, RTX, MDZ	NR	Gradual recovery
		Kadoya M. *et al* [81]	1	2015	Japan	48	1w	LEV, PB, TOP, LMG, PPF, MDZ, MTP, PLPH, IVIG, RTX, CyC	Yes	Complete recovery in 7m
		Gaig C. *et al* [82]	1	2014	Spain	26	<1d	LEV, VPA, PHT LCM, MDZ, PPF, THP	Yes	Partial recovery in 2m
		Johnson N. *et al* [83]	1	2010	USA	35	3w	PHT, VPA, LEV, BZD, PPF, PTB, IVIG, RTX, CyC, tumor removal	Yes	Partial recovery in 1y
		Kirkpatrick MP. *et al* [84]	1	2010	USA	19	3m	LOR, DZP, PHT, LEV, VPA, PPF, RTX, STE, PLPH, FBT, tumor removal	Yes	Partial recovery in 6w
	Other encephalopathy responsive to immunotherapy	Gaig C. *et al* [82]	1	2014	Spain	29	<1d	CLZ, LEV, VPA, LCM, PPF, MDZ, THP	Yes	Partial recovery

Metabolic	Acute porphyria	Dawit S. *et al* [85]	1	2018	USA	71	NR	LEV, Hemin	NR	Complete recovery
	Citrullinemia	Funabe S. *et al* [86]	1	2009	Japan	47	NR	DZP, arginine, PHT, CBZ, liver transplant	No	Complete recovery
	Chronic renal failure	Linetsky E. *et al* [87]	1	2000	Israel	65	NR	DZP, PHT	NR	Complete recovery in <1d
		Korn-Lubetzki I. *et al* [78]	3	2007	Israel	88	3d	DZP, PHT, VPA	No	Complete recovery in 3d
		Pollock LM. *et al* [88]	1	2000	UK	67	10d	DZP, VPA	NR	Died in 23d
	Decompensated diabetes	Tedrus GM. *et al* [89]	1	1997	Brazil	52	1d	DZP, VPA	NR	Complete recovery
	Hashimoto's encephalopathy	Monti G. *et al* [90]	2	2011	Italy	59	NR (n=2)	DZP, PHT, LOR (n=1)<br>VPA, MTP (n=2)	NR (n=2)	Complete recovery in 2d
	Hemodialysis	Iftikhar S. *et al* [13]	1	2007	Saudi Arabia	75	1d	DZP	NR	Complete recovery in 1d
	Hepatic encephalopathy	Jo YM. *et al* [91]	1	2015	Korea	52	2d	LOR, LEV	NR	Died in 11d
		Gaig C. *et al* [82]	1	2014	Spain	62	2d	LEV, PHT, MDZ PPF, THP	Yes	Died in 26d
		Kaplan PW. *et al* [92]	1	1996	USA	71	<1d	DZP, PHT	NR	Recovery in 2d
	Hyperosmolar state	Kutluay E. *et al* [93]	1	2007	Turkey	57	4d	PHT, MDZ	NR	Complete recovery in 6h
		Thomas P. *et al* [94]	1	1997	France	60	NR	PHT, CBZ	NR	Complete recovery
	Hypomagnesemia	Uthman B. *et al* [56]	1	2007	USA	85	2d	LOR, VPA, PPF	yes	Died in 7w
	Hyponatremia	Liang CS. *et al* [95]	1	2011	Taiwan	64	7d	LEV, VPA, RPD	No	Complete recovery in 4d
		Bottaro FJ. *et al* [96]	9	2007	Argentina	81	NR	PHT (n=4)<br>PHT, VAL (n=3)	NR	Complete recovery (n=2)

								VAL (n=2)		Partial recovery (n=4)<br>Died (n=3)
	Metabolic disarrangement	Labar D. *et al* [97]	2	1998	USA	80	NR	NR	NR	NR**
	Hypothyroidism	Rocco M. *et al* [98]	1	2011	Italy	73	<1d	DZP, PHT, PPF, T3	Yes	Complete recovery in 1w
Encephalitis	Unknown	Fernández-Torre JL. *et al* [99]	2	2012	Spain	47	NR (n=2)	PHT, CLZ, VPA, PPF (n=2)<br>MDZ, PB, THP, STE (n=1)	Yes (n=2)	Complete recovery (n=1)<br>Died (n=1)
		Veran O. *et al* [100]	1	2010	France	73	NR	NR	NR	Partial recovery
Malignancy	Cerebral tumor	Gaig C. *et al* [82]	1	2014	Spain	80	<1d	VPA, LEV, MDZ PPF, THP	Yes	Died in 15d
		Labar d. *et al* [97]	1	1998	USA	80	NR	NR	NR	NR**
	Endometrial cancer	Yilmaz A. *et al* [101]	1	2003	Turkey	62	<1d	PHT, DZP, MDZ	Yes	Died in 5d
	Glioblastoma	Chang JW. *et al* [102]	1	2001	Korea	60	NR	CBZ, VPA	5d	Complete recovery in 15w
	Leptomeningeal cancer	Broderick JP. *et al* [103]	1	1987	USA	56	<1d	DZP, PHT	NR	Complete recovery in <1d
	Lung cancer	Miranda M. *et al* [104]	1	2010	Chile	68	<1d	PHT, MDZ, PPF, TOP, LEV	Yes	Recovery

	Melanoma, breast, CML and lung cancer	Narayanan JT. *et al* [105]	4	2006	USA	62	NR	PPH, LOR (n=4) VPA (n=1)	NR (n=4)	Complete recovery (n=2) Partial recovery (n=1) Died in 5d (n=1)
	Prostate cancer	Samala RV. *et al* [106]	1	2015	USA	66	3d	LEV, LCM	No	Died in a few days
	Renal cancer	Lorenzl S. *et al* [107]	1	2008	Germany	52	1w	LOR, PHT, VPA, MDZ, LEV	NR	Died in 7d
Neurovascular	Bitemporal hypoperfusion	Hasegawa T. *et al* [108]	1	2005	Japan	78	7m	DZP, CBZ, CLZ	NR	Complete recovery
	Cerebral vasogenic edema	Kim TE. *et al* [109]	1	2012	Korea	74	5d	LOR, VPA	No	Complete recovery in 2d
	Focal neurological deficit	Hilkens PHE. *et al* [44]	17	1995	The Netherlands	72	4d	CLZ and PHT or CBZ (n=4)	NR	Complete recovery in a median of 5d (n=10) Partial recovery in a median of 10d (=4) Non recovery (n=3)
	Ictal psychosis	López Arteaga T. *et al* [110]	1	2013	Spain	31	2d	RPD, LOR, HPD, DZP	No	Complete recovery in 1d
	Ischemic stroke	Schomer AC. *et al* [111]	1	2015	USA	80	2d	LEV	NR	Complete recovery in 1d
		Wakisaka K. *et al* [112]	1	2015	Japan	74	4d	PPH, CBZ	NR	Complete recovery in 5d
	Reversible cerebellar lesion	Ohe Y. *et al* [113]	1	2013	Japan	67	<1d	VPA, DZP, LEV	NR	Complete recovery in 11d

	Sinus thrombosis	Hilkens PHE. *et al* [44]	1	1995	The Netherlands	73	<1d	CLZ, PHT	NR	Partial recovery in 3m
	SIRPID phenomenon	Zeiler SR. *et al* [114]	1	2011	USA	62	<1d	LOR, LEV, PHT, PPH	Yes	Partial recovery in weeks
	Stroke	Arif H. *et al* [79]	1	2008	USA	67	<1d	PPH, LOR, LEV, GBP		
		Labar D. *et al* [97]	4	1998	USA	80	NR	NR	NR	NR**
		Veran O. et al [100]	3	2010	France	75	NR	NR	NR	Complete recovery
		Fernandez-Torre JL *et al* [115]	1	2007	Spain	88	3d	PHT, VPA, CLZ	NR	Partial recovery in 23d
		Fernández-Torre JL. *et al* [6]	7	2012	Spain	77	1d	PHT, VPA, PPF, PPH, CLZ, PPF	NR	Partial recovery (N=4)<br>Died (N=3)
	Acute or previous stroke	Canas N. *et al* [116]	14	2018	Portugal	76	NR	PHT, LEV. VPA	NR*	NR*
	Subdural hematoma	Fernández-Torre JL. *et al* [6]	2	2012	Spain	72	1d	PHT, VPA	NR	Died (n=1)<br>Partial recovery in 7d (n=1)
	Temporo-occipital stroke	Sethi NK. *et al [11]*	1	2010	USA	93	1d	LEV, PHT, VPA	Yes	Died in 25d
Other causes	Cerebral fat embolism	Chatterjee R. *et al* [117]	1	2017	India	22	NR	MDZ, LEV, VPA, LCM	Yes	Complete recovery in 8d
		Fernández-Torre J.L. *et al* [118]	1	2015	Spain	82	2d	LEV, PFP, MDZ, VPA, LCM	Yes	Died in 21d
	New-onset seizure disorder	Derakhshan I. *et al* [119]	1	2006	USA	77	<1d	DZP, TOP	NR	Complete recovery in <1d

		Riggio S. *et al* [71]	3	2005	USA	69	<2d	DZP, PHT (n=2)<br>PTB (n=1)<br>LOR (n=1)	NR (n=3)	Recovery in <1d (n=1)<br>Recovery (n=2)
	Postoperative blindness	Ibrahim TF. *et al* [120]	1	2017	USA	67	<1d	LEV, LCM, LOR, MDZ	Yes	Complete recovery in 5d
	Pupillary hippos	Fernández-Torre JL. *et al* [121]	1	2018	Spain	52	2d	PHT, LEV, VPA, LCM, DXM	Yes	Died
	PRES	Bhatt A. *et al* [122]	1	2009	USA	45	8d	PHT, LOR	Yes	Complete recovery in 3m
	Small-bowel obstruction	Riggio S. *et al* [123]	1	2006	USA	68	NR	LOR	NR	Recovery
	Temporal mesial sclerosis	Warraich S. *et al* [124]	1	2018	Kingdom of Bahrain	29	1w	DZP, PHT, PPF, LEV	Yes	Complete recovery in 8d
	Hypothermia	Korn-Lubetzki I. *et al* [78]	1	2007	Israel	73	1d	DZP, PHT, VPA	No	Complete recovery in 4d
	Thrombotic Thrombocytopenic Purpura	Blum AS. *et al* [125]	1	1996	USA	87	<1d	PHT, LOR	NR	Died in 1w
		Garret WT. *et al* [126]	1	1996	USA	52	2d (n=2)	PHT, DZP, PB, PLPH (n=2)<br>VPA, CBZ (n=1)	NR (n=2)	Partial recovery (n=1)<br>Died (n=1)
Other medical conditions	Cardiovascular-diseases	Bottaro FJ. *et al* [96]	5	2007	Argentina	85	NR	PHT (n=1)<br>PHT, VAL (n=1)<br>VAL (n=3)	NR	Total recovery (n=1)<br>Partial recovery (n=3)<br>Died (n=1)
	Anoxia	Fernández-Torre JL. *et al* [6]	3	2012	Spain	72	1d	PHT, MDZ, PPF	NR	Died (n=2)<br>Partial recovery (n=1)
	Cryptogenic	Bottaro FJ. *et al* [96]	3	2007	Argentina	85	NR	PHT (n=2)<br>PHT, VAL, LEV (n=1)	NR	Complete recovery (n=1)<br>No recovery (n=1)

										Died (n=1)
		Veran O. *et al* [100]	2	2010	France	66	NR	NR	NR	Complete recovery
		Canas N. *et al* [116]	4	2018	Portugal	76	NR	PHT, LEV, VPA	NR*	NR*
		Fernández-Torre JL. *et al* [6]	2	2012	Spain	75	1d	LEV, VPA, CLZ, PHT	NR	Complete recovery
	Metabolic or infectious causes	Canas N. *et al* [116]	22	2018	Portugal	76	NR	PHT, LEV, VPA	NR*	NR*
	Previous brain damage	Labar D. *et al* [97]	3	1998	USA	80	NR	NR	NR	NR**
Psychogenic	Capgras syndrome	Turtzo L. *et al* [127]	1	2008	USA	87	>3d	PHT, LEV, LMG	NR	Recovery
	Cortical epilepsy	Lim J. *et al* [128]	3	1985	USA	60	<1d (n=2) 3d (n=1)	PHT (n=3)	NR (n=3)	Complete recovery in <1d (n=3)
	Frontal NCSE	Chicharro-Ciuffardi A. *et al* [129]	1	2012	Chile	20	2m	LMG	No	Complete recovery
		Kaplan PW. *et al* [130]	1	2011	USA	73	Several days	BZD, LEV, VPA	NR	Partial recovery in days
		Takaya S. *et al* [131]	1	2004	Japan	69	<1d	CBZ, VPA	NR	Complete recovery in 1m
	Occipital NCSE	Chu K. *et al* [132]	1	2001	South Korea	56	3d	DZP, LOR, PHT, MDZ, VPA, DPH	NR	Not recovery
	Temporal NCSE	Chung PW. *et al* [133]	1	2002	Korea	62	5w	DPH	NR	Complete recovery in 4m

	Temporo-parietal NCSE	Flacke S. *et al* [134]	1	1999	Germany	68	3h	CLZ, MPHT	NR	Complete recovery in <1d
Possible diagnostic pitfall	UTI (n=8)<br>ACS (n=2)<br>Refusal to eat (n=4)	Shavit L. *et al* [135]	13	2012	Israel	81	2d (n=13)	PHT (n=10)<br>VPA (n=4)	NR (n=13)	Complete recovery (n=5)<br>Partial recovery (n=5)<br>Died (n=3)
Cognitive impairment	Dementia	Korn-Lubetzki I. *et al* [78]	2	2007	Israel	80	2d	DZP, PHY. VPA	No	Complete recovery in 2 d
		Veran O. *et al* [100]	1	2010	France	97	NR	NR	NR	Died

NP: not performed, NR: not reported, D: day, W: week, Min: minutes, LEV: levetiracetam, LCM: lacosamide, PHT: Phenytoin, MPHT: mephenytoin, VPA: Valproic acid, BZD: benzodiazepines, PB: phenobarbital, DXM: dexamethasone, DZP: diazepam, THP: thiopental, PPF: propofol, PPH: phospho-phenytoin, MDZ: midazolam, LMG: lamotrigine, RPD: risperidone, HPD: Haloperidol, MTP: methylprednisolone, PDN: prednisone, CyC: cyclophosphamide, IVIG: immunoglobulins, RTX: rituximab, CLZ: clonazepam, CLP: chlorpromazine , QCN: quinacrine, PLPH: plasmapheresis, ZND: zonisamide, FBT: felbamate, PTB: pentobarbital, GBP: gabapentin, DPH: diphenylhydantoin, STE: steroids, NLX: naloxone, (ISN + RIF + PYR + ETB): isoniazid, rifampicin, pyrazinamide and ethambutol, SIRPID: stimulus-induced rhythmic, periodic, or ictal discharges, PRES: posterior reversible encephalopathy syndrome, UTI: urinary tract infection, ACS: acute coronary syndrome. *Global data from Canas N. *et al*: 6 patients admitted in ICU, 9 patients died. **Global data from Labar D. *et al*: 3 patients died and 2 presented a complete recovery.

References

1. Cunningham JM, Sachs KV, Allyn R. Cefepime-Induced Neurotoxicity Presenting with Nonconvulsive Status Epilepticus Admitted as a Stroke Alert. Am J Case Rep. 2020;21: e921643. doi:10.12659/AJCR.921643

2. Tchapyjnikov D, Luedke MW. Cefepime-Induced Encephalopathy and Nonconvulsive Status Epilepticus: Dispelling an Artificial Dichotomy. The Neurohospitalist. 2019;9: 100–104. doi:10.1177/1941874418803225

3. Skaistys A, Rätz Bravo AE, Leuppi-Taegtmeyer A, Zysset Y. [Fatal Outcome of Agranulocytosis after Re-Exposure to Metamizole and Cefepime-Induced Encephalopathy]. Praxis. 2019;108: 693–697. doi:10.1024/1661-8157/a003275

4. Hagiya H, Miyawaki K, Yamamoto N, Yoshida H, Kitagawa A, Asaoka T, et al. Ceftriaxone-induced Neurotoxicity in a Patient after Pancreas-Kidney Transplantation. Intern Med Tokyo Jpn. 2017;56: 3103–3107. doi:10.2169/internalmedicine.8774-16

5. Collins RD, Tverdek FP, Bruno JJ, Coyle EA. Probable Nonconvulsive Status Epilepticus With the Use of High-Dose Continuous Infusion Ceftazidime. J Pharm Pract. 2016;29: 564–568. doi:10.1177/0897190015608503

6. Fernández-Torre JL, Rebollo M, Gutiérrez A, López-Espadas F, Hernández-Hernández MA. Nonconvulsive status epilepticus in adults: electroclinical differences between proper and comatose forms. Clin Neurophysiol Off J Int Fed Clin Neurophysiol. 2012;123: 244–251. doi:10.1016/j.clinph.2011.06.020

7. Balderia PG, Chandorkar A, Kim Y, Patnaik S, Sloan J, Newman GC. Dosing Cefepime for Renal Function Does Not Completely Prevent Neurotoxicity in a Patient With Kidney Transplant. J Patient Saf. 2018;14: e33–e34. doi:10.1097/PTS.0000000000000225

8. Kim A, Kim J-E, Paek Y-M, Hong K-S, Cho Y-J, Cho J-Y, et al. Cefepime- Induced Non-Convulsive Status Epilepticus (NCSE). J Epilepsy Res. 2013;3: 39–41. doi:10.14581/jer.13008

9. Chang Y-M. Cefepime-induced nonconvulsive status epilepticus as a cause of confusion in an elderly patient. J Formos Med Assoc Taiwan Yi Zhi. 2015;114: 290–291. doi:10.1016/j.jfma.2013.05.002

10. Kim KB, Kim SM, Park W, Kim JS, Kwon SK, Kim H-Y. Ceftiaxone-induced neurotoxicity: case report, pharmacokinetic considerations, and literature review. J Korean Med Sci. 2012;27: 1120–1123. doi:10.3346/jkms.2012.27.9.1120

11. Sethi NK, Torgovnick J, Sethi PK, Arsura E. Nonconvulsive status epilepticus presenting with throat clearing as part of clinical seizure semiology. Clin EEG Neurosci. 2010;41: 50–52. doi:10.1177/155005941004100110

12. Spriet I, Meersseman W, De Troy E, Wilmer A, Casteels M, Willems L. Meropenem - valproic acid interaction in patients with cefepime-associated status epilepticus. Am J Health-Syst Pharm AJHP Off J Am Soc Health-Syst Pharm. 2007;64: 54–58. doi:10.2146/ajhp050512

13. Iftikhar S, Dahbour S, Nauman S. Nonconvulsive status epilepticus: high incidence in dialysis-dependent patients. Hemodial Int Int Symp Home Hemodial. 2007;11: 392–397. doi:10.1111/j.1542-4758.2007.00206.x

14. Vannaprasaht S, Tawalee A, Mayurasakorn N, Yodwut C, Bansong R, Reungjui S, et al. Ceftazidime overdose-related nonconvulsive status epilepticus after intraperitoneal instillation. Clin Toxicol Phila Pa. 2006;44: 383–386. doi:10.1080/15563650600671753

15. Maganti R, Jolin D, Rishi D, Biswas A. Nonconvulsive status epilepticus due to cefepime in a patient with normal renal function. Epilepsy Behav EB. 2006;8: 312–314. doi:10.1016/j.yebeh.2005.09.010

16. Primavera A, Cocito L, Audenino D. Nonconvulsive status epilepticus during cephalosporin therapy. Neuropsychobiology. 2004;49: 218–222. doi:10.1159/000077370

17. Abanades S, Nolla J, Rodríguez-Campello A, Pedro C, Valls A, Farré M. Reversible coma secondary to cefepime neurotoxicity. Ann Pharmacother. 2004;38: 606–608. doi:10.1345/aph.1D322

18. Plensa E, Gallardo E, Ribera J-M, Batlle M, Oriol A, Costa J. Nonconvulsive status epilepticus associated with cefepime in a patient undergoing autologous stem cell transplantation. Bone Marrow Transplant. 2004;33: 119–120. doi:10.1038/sj.bmt.1704314

19. Ferrara N, Abete P, Giordano M, Ferrara P, Carnovale V, Leosco D, et al. Neurotoxicity induced by Cefepime in a very old hemodialysis patient. Clin Nephrol. 2003;59: 388–390. doi:10.5414/cnp59388

20. Chow KM, Wang AY, Hui AC, Wong TY, Szeto CC, Li PK. Nonconvulsive status epilepticus in peritoneal dialysis patients. Am J Kidney Dis Off J Natl Kidney Found. 2001;38: 400–405. doi:10.1053/ajkd.2001.26105

21. Chatellier D, Jourdain M, Mangalaboyi J, Ader F, Chopin C, Derambure P, et al. Cefepime-induced neurotoxicity: an underestimated complication of antibiotherapy in patients with acute renal failure. Intensive Care Med. 2002;28: 214–217. doi:10.1007/s00134-001-1170-9

22. Martínez-Rodríguez JE, Barriga FJ, Santamaria J, Iranzo A, Pareja JA, Revilla M, et al. Nonconvulsive status epilepticus associated with cephalosporins in patients with renal failure. Am J Med. 2001;111: 115–119. doi:10.1016/s0002-9343(01)00767-7

23. Saurina A, Vera M, Pou M, López Pedret J, Darnell A, Campistol JM, et al. [Non-convulsive status epilepticus secondary to adjusted cefepime doses in patients with chronic renal failure]. Nefrol Publicacion Of Soc Espanola Nefrol. 2000;20: 554–558.

24. Dixit S, Kurle P, Buyan-Dent L, Sheth RD. Status epilepticus associated with cefepime. Neurology. 2000;54: 2153–2155. doi:10.1212/wnl.54.11.2153

25. Klion AD, Kallsen J, Cowl CT, Nauseef WM. Ceftazidime-related nonconvulsive status epilepticus. Arch Intern Med. 1994;154: 586–589.

26. Rissardo JP, Caprara ALF, Silveira JOF. Generalized Convulsive Status Epilepticus Secondary to Jarisch-Herxheimer Reaction in Neurosyphilis: A Case Report and Literature Review. The Neurologist. 2019;24: 29–32. doi:10.1097/NRL.0000000000000219

27. Gürses C, Kürtüncü M, Jirsch J, Yeşilot N, Hanağasi H, Bebek N, et al. Neurosyphilis presenting with status epilepticus. Epileptic Disord Int Epilepsy J Videotape. 2007;9: 51–56. doi:10.1684/epd.2007.0058

28. Kojan S, Van Ness PC, Diaz-Arrastia R. Nonconvulsive status epilepticus resulting from Jarisch-Herxheimer reaction in a patient with neurosyphilis. Clin EEG Electroencephalogr. 2000;31: 138–140. doi:10.1177/155005940003100306

29. Zifko U, Lindner K, Wimberger D, Volc B, Grisold W. Jarisch-Herxheimer reaction in a patient with neurosyphilis. J Neurol Neurosurg Psychiatry. 1994;57: 865–867. doi:10.1136/jnnp.57.7.865

30. Holman LL, Ren Y, Westin SN. Status epilepticus associated with platinum chemotherapy in a patient with cervical cancer: a case report. BMC Cancer. 2015;15: 728. doi:10.1186/s12885-015-1755-2

31. Palma J-A, Gomez-Ibañez A, Martin B, Urrestarazu E, Gil-Bazo I, Pastor MA. Nonconvulsive status epilepticus related to posterior reversible leukoencephalopathy syndrome induced by cetuximab. The Neurologist. 2011;17: 273–275. doi:10.1097/NRL.0b013e3182173655

32. Taupin D, Racela R, Friedman D. Ifosfamide chemotherapy and nonconvulsive status epilepticus: case report and review of the literature. Clin EEG Neurosci. 2014;45: 222–225. doi:10.1177/1550059413500777

33. Kilickap S, Cakar M, Onal IK, Tufan A, Akoglu H, Aksoy S, et al. Nonconvulsive status epilepticus due to ifosfamide. Ann Pharmacother. 2006;40: 332–335. doi:10.1345/aph.1G363

34. Bhardwaj A, Badesha PS. Ifosfamide-induced nonconvulsive status epilepticus. Ann Pharmacother. 1995;29: 1237–1239. doi:10.1177/106002809502901210

35. Wengs WJ, Talwar D, Bernard J. Ifosfamide-induced nonconvulsive status epilepticus. Arch Neurol. 1993;50: 1104–1105. doi:10.1001/archneur.1993.00540100089024

36. Yoshino A, Watanabe M, Shimizu K, Goto T, Ichinowatari N, Yoshimasu H, et al. Nonconvulsive status epilepticus during antidepressant treatment. Neuropsychobiology. 1997;35: 91–94. doi:10.1159/000119397

37. Zak R, Solomon G, Petito F, Labar D. Baclofen-induced generalized nonconvulsive status epilepticus. Ann Neurol. 1994;36: 113–114. doi:10.1002/ana.410360122

38. Benbadis SR, Van Ness PC. Chloroquine and nonconvulsive status epilepticus. Ann Intern Med. 1996;124: 614–615. doi:10.7326/0003-4819-124-6-199603150-00020

39. Pogliani D, Pozzi A, Laudi C, Rimoldi L, Sogni E, Farfaglia P, et al. [Nonconvulsive status epilepticus due to Fentanyl intoxication in hemodialysed patients: two case reports and review of the literature]. G Ital Nefrol Organo Uff Della Soc Ital Nefrol. 2016;33.

40. Roccatagliata L, Audenino D, Primavera A, Cocito L. Nonconvulsive status epilepticus from accidental lithium ingestion. Am J Emerg Med. 2002;20: 570–572. doi:10.1053/ajem.2002.34957

41. Patterson DM, Aries J, Hyare H, Holder D, Rees J, Lee SM. Nonconvulsive status epilepticus and leucoencephalopathy after high-dose methotrexate. J Clin Oncol Off J Am Soc Clin Oncol. 2011;29: e459-461. doi:10.1200/JCO.2010.33.9598

42. Bertran F, Denise P, Letellier P. Nonconvulsive status epilepticus: the role of morphine and its antagonist. Neurophysiol Clin Clin Neurophysiol. 2000;30: 109–112. doi:10.1016/s0987-7053(00)00059-9

43. Krieger AC, Takeyasu M. Nonconvulsive status epilepticus in theophylline toxicity. J Toxicol Clin Toxicol. 1999;37: 99–101. doi:10.1081/clt-100102414

44. Hilkens PH, de Weerd AW. Non-convulsive status epilepticus as cause for focal neurological deficit. Acta Neurol Scand. 1995;92: 193–197. doi:10.1111/j.1600-0404.1995.tb01687.x

45. Mogi T, Toda H, Tatsuzawa Y, Fukutomi T, Soga S, Shinmoto H, et al. Clinically mild encephalopathy with a reversible splenial lesion and nonconvulsive status epilepticus in a schizophrenic patient with neuroleptic malignant syndrome. Psychiatry Clin Neurosci. 2017;71: 212. doi:10.1111/pcn.12492

46. Yoshino A, Yoshimasu H. Nonconvulsive status epilepticus complicating neuroleptic malignant syndrome improved by intravenous diazepam. J Clin Psychopharmacol. 2000;20: 389–390. doi:10.1097/00004714-200006000-00020

47. Olnes MJ, Golding A, Kaplan PW. Nonconvulsive status epilepticus resulting from benzodiazepine withdrawal. Ann Intern Med. 2003;139: 956–958. doi:10.7326/0003-4819-139-11-200312020-00022

48. Fernández-Torre JL. De novo absence status of late onset following withdrawal of lorazepam: a case report. Seizure. 2001;10: 433–437. doi:10.1053/seiz.2000.0510

49. Kanemoto K, Miyamoto T, Abe R. Ictal catatonia as a manifestation of de novo absence status epilepticus following benzodiazepine withdrawal. Seizure. 1999;8: 364–366. doi:10.1053/seiz.1999.0309

50. Primavera A, Cocito L. Acute confusion in a chronic benzodiazepine patient. Withdrawal-related nonconvulsive status epilepticus misdiagnosed as acute intoxication. Gen Hosp Psychiatry. 1995;17: 460–462. doi:10.1016/0163-8343(95)90050-0

51. Vickrey BG, Bahls FH. Nonconvulsive status epilepticus following cerebral angiography. Ann Neurol. 1989;25: 199–201. doi:10.1002/ana.410250217

52. Amer-Ferrer G, Alonso-Marné T, Diez-Tejedor E, Rodriguez-Albariño A, Frank A, Barreiro-Tella P. Absence status following metrizamide myelography: management of nonresponders. Eur Neurol. 1988;28: 341–344. doi:10.1159/000116298

53. Levin R, Lee SI. Nonconvulsive status epilepticus following metrizamide myelogram. Ann Neurol. 1985;17: 518–519. doi:10.1002/ana.410170519

54. Pritchard PB, O'Neal DB. Nonconvulsive status epilepticus following metrizamide myelography. Ann Neurol. 1984;16: 252–254. doi:10.1002/ana.410160216

55. Brown KL, Wilson RF, White MT. Carbon monoxide-induced status epilepticus in an adult. J Burn Care Res Off Publ Am Burn Assoc. 2007;28: 533–536. doi:10.1097/BCR.0B013E318053DA82

56. Uthman B, Bearden S. Rhythmic diffuse delta frequency activity presenting as an unusual EEG correlate of nonconvulsive status epilepticus: three case studies. Epilepsy Behav EB. 2008;12: 191–199. doi:10.1016/j.yebeh.2007.08.019

57. Fernández-Torre JL, Paramio-Paz A, Rodríguez-Borregán JC, Orizaola P, Bosque-Varela P, Hernández-Hernández MA. Super-refractory nonconvulsive status epilepticus due to self-poisoning with metaldehyde. J Clin Neurosci Off J Neurosurg Soc Australas. 2018;47: 134–136. doi:10.1016/j.jocn.2017.10.017

58. Denays R, Kumba C, Lison D, De Bels D. First epileptic seizure induced by occupational nickel poisoning. Epilepsia. 2005;46: 961–962. doi:10.1111/j.1528-1167.2005.70004.x

59. Kaplan PW, Billnitzer A, Fernández-Torre JL. Subacute Encephalopathy With Seizures in Alcoholics (SESA) Presenting as Focal Nonconvulsive Status Epilepticus. Clin EEG Neurosci. 2018;49: 414–416. doi:10.1177/1550059417747435

60. Fernández-Torre JL, Kaplan PW. Subacute encephalopathy with seizures in alcoholics (SESA syndrome) revisited. Seizure. 2014;23: 393–396. doi:10.1016/j.seizure.2014.02.002

61. Wong MC, Suite ND, Labar DR. Nonconvulsive generalized status epilepticus and AIDS. Ann Intern Med. 1992;116: 171–172. doi:10.7326/0003-4819-116-2-171

62. Sharma DK, Boggild M, van Heuven AW, White RP. Creutzfeldt-Jakob Disease Presenting as Stroke: A Case Report and Systematic Literature Review. The Neurologist. 2017;22: 48–53. doi:10.1097/NRL.0000000000000107

63. Albanese M, Placidi F, Romigi A, Schirinzi T, Liguori C, Marchi A, et al. Symptomatic nonconvulsive status epilepticus erroneously suggestive of sporadic Creutzfeldt-Jakob disease. J Neurol Sci. 2015;348: 274–276. doi:10.1016/j.jns.2014.11.012

64. Aiguabella M, Falip M, Veciana M, Bruna J, Palasí A, Corral L, et al. Refractory nonconvulsive status epilepticus in Creutzfeldt-Jakob disease. Epileptic Disord Int Epilepsy J Videotape. 2010;12: 239–242. doi:10.1684/epd.2010.0318

65. Espinosa PS, Bensalem-Owen MK, Fee DB. Sporadic Creutzfeldt-Jakob disease presenting as nonconvulsive status epilepticus case report and review of the literature. Clin Neurol Neurosurg. 2010;112: 537–540. doi:10.1016/j.clineuro.2010.03.025

66. Rossetti AO, Dunand M. Creutzfeldt-Jakob disease: evolution from nonconvulsive status epilepticus, through SIRPIDs, to generalized periodic discharges. Clin Neurophysiol Off J Int Fed Clin Neurophysiol. 2007;118: 2533–2536. doi:10.1016/j.clinph.2007.08.002

67. Cohen D, Kutluay E, Edwards J, Peltier A, Beydoun A. Sporadic Creutzfeldt-Jakob disease presenting with nonconvulsive status epilepticus. Epilepsy Behav EB. 2004;5: 792–796. doi:10.1016/j.yebeh.2004.06.019

68. Shapiro JM, Shujaat A, Wang J, Chen X. Creutzfeldt-Jakob disease presenting as refractory nonconvulsive status epilepticus. J Intensive Care Med. 2004;19: 345–348. doi:10.1177/0885066604269771

69. Cury RF, Wichert-Ana L, Sakamoto AC, Fernandes RMF. Focal nonconvulsive status epilepticus associated to PLEDs and intense focal hyperemia in an AIDS patient. Seizure. 2004;13: 358–361. doi:10.1016/j.seizure.2003.07.001

70. Assenza F, Tombini M, Assenza G, Campana C, Benvenga A, Brunelli N, et al. Dengue encephalitis presenting with nonconvulsive status epilepticus: A case report. Clin Neurol Neurosurg. 2016;150: 89–91. doi:10.1016/j.clineuro.2016.08.027

71. Riggio S. Nonconvulsive status epilepticus: clinical features and diagnostic challenges. Psychiatr Clin North Am. 2005;28: 653–664, 662. doi:10.1016/j.psc.2005.05.003

72. Lee J-H, Nam D-H, Oh S-Y, Shin B-S, Seo M-W, Jeong S-K, et al. Nonconvulsive status epilepticus presenting as epileptic nystagmus in a patient with herpes encephalitis. J Neuro-Ophthalmol Off J North Am Neuro-Ophthalmol Soc. 2012;32: 249–251. doi:10.1097/WNO.0b013e3182413679

73. Primavera A, Solaro C, Cocito L. De novo status epilepticus as the presenting sign of neurosyphilis. Epilepsia. 1998;39: 1367–1369. doi:10.1111/j.1528-1157.1998.tb01339.x

74. Wusthoff CJ, Kranick SM, Morley JF, Christina Bergqvist AG. The ketogenic diet in treatment of two adults with prolonged nonconvulsive status epilepticus. Epilepsia. 2010;51: 1083–1085. doi:10.1111/j.1528-1167.2009.02388.x

75. Arman F, Kaya D, Akgün Y, Kocagöz S. Tuberculous meningitis presenting with nonconvulsive status epilepticus. Epilepsy Behav EB. 2011;20: 111–115. doi:10.1016/j.yebeh.2010.10.014

76. Shea Y, Cheng K, Lee C, Chang SR, Yau ZS. Refusal to Eat as the Initial Manifestation of Nonconvulsive Status Epilepticus-Need for Clinical Vigilance. J Am Geriatr Soc. 2016;64: 239–240. doi:10.1111/jgs.13905

77. Lin Y-J, Lo C, Cheng S-J, Chou C-L, Hseuh I-H. Recurrent nonconvulsive status epilepticus in a patient with progressive left hemispheric leukoencephalopathy after a remote viral meningoencephalitis. Epilepsy Behav EB. 2015;49: 178–183. doi:10.1016/j.yebeh.2015.05.023

78. Korn-Lubetzki I, Steiner-Birmanns B, Galperin I, Benasouli Y, Steiner I. Nonconvulsive status epilepticus in older people: a diagnostic challenge and a treatable condition. J Am Geriatr Soc. 2007;55: 1475–1476. doi:10.1111/j.1532-5415.2007.01284.x

79. Arif H, Hirsch LJ. Treatment of status epilepticus. Semin Neurol. 2008;28: 342–354. doi:10.1055/s-2008-1079339

80. Shi Y. Serial EEG Monitoring in a Patient With Anti-NMDA Receptor Encephalitis. Clin EEG Neurosci. 2017;48: 301–303. doi:10.1177/1550059416677398

81. Kadoya M, Onoue H, Kadoya A, Ikewaki K, Kaida K. Refractory status epilepticus caused by anti-NMDA receptor encephalitis that markedly improved following combination therapy with rituximab and cyclophosphamide. Intern Med Tokyo Jpn. 2015;54: 209–213. doi:10.2169/internalmedicine.54.2047

82. Gaig C, Iranzo A, Tercero A, Herman ST, Santamaria J. Stimulus-induced generalized epileptiform discharges: an unrecognized EEG pattern in refractory nonconvulsive status epilepticus. J Clin Neurophysiol Off Publ Am Electroencephalogr Soc. 2014;31: 580–585. doi:10.1097/WNP.0000000000000092

83. Johnson N, Henry C, Fessler AJ, Dalmau J. Anti-NMDA receptor encephalitis causing prolonged nonconvulsive status epilepticus. Neurology. 2010;75: 1480–1482. doi:10.1212/WNL.0b013e3181f8831a

84. Kirkpatrick MP, Clarke CD, Sonmezturk HH, Abou-Khalil B. Rhythmic delta activity represents a form of nonconvulsive status epilepticus in anti-NMDA receptor antibody encephalitis. Epilepsy Behav EB. 2011;20: 392–394. doi:10.1016/j.yebeh.2010.11.020

85. Dawit S, Bhatt SK, Das DM, Pines AR, Shiue HJ, Goodman BP, et al. Nonconvulsive status epilepticus secondary to acute porphyria crisis. Epilepsy Behav Case Rep. 2019;11: 43–46. doi:10.1016/j.ebcr.2018.11.002

86. Funabe S, Tanaka R, Urabe T, Kawasaki S, Kobayashi K, Hattori N. [A case of adult-onset type II citrullinemia with repeated nonconvulsive status epilepticus]. Rinsho Shinkeigaku. 2009;49: 571–575. doi:10.5692/clinicalneurol.49.571

87. Linetsky E, Planer D, Ben-Hur T. Echolalia-palilalia as the sole manifestation of nonconvulsive status epilepticus. Neurology. 2000;55: 733–734. doi:10.1212/wnl.55.5.733

88. Pollock LM, Mitchell SC. Nonconvulsive status epilepticus causing acute confusion. Age Ageing. 2000;29: 360–362. doi:10.1093/ageing/29.4.360

89. Tedrus GM, Fonseca LC, De Tella LM, Viana MA. [De novo absence status: case report]. Arq Neuropsiquiatr. 1997;55: 642–645. doi:10.1590/s0004-282x1997000400022

90. Monti G, Pugnaghi M, Ariatti A, Mirandola L, Giovannini G, Scacchetti S, et al. Non-convulsive status epilepticus of frontal origin as the first manifestation of Hashimoto's encephalopathy. Epileptic Disord Int Epilepsy J Videotape. 2011;13: 253–258. doi:10.1684/epd.2011.0457

91. Jo YM, Lee SW, Han SY, Baek YH, Ahn JH, Choi WJ, et al. Nonconvulsive status epilepticus disguising as hepatic encephalopathy. World J Gastroenterol. 2015;21: 5105–5109. doi:10.3748/wjg.v21.i16.5105

92. Kaplan PW. Nonconvulsive status epilepticus in the emergency room. Epilepsia. 1996;37: 643–650. doi:10.1111/j.1528-1157.1996.tb00628.x

93. Kutluay E, Pakoz B, Yuksel A, Beydoun A. Nonconvulsive status epilepticus manifesting as pure alexia (alexia without agraphia). Epilepsy Behav EB. 2007;10: 626–628. doi:10.1016/j.yebeh.2007.03.001

94. Thomas P, Mottin Y. [Simple partial frontal nonconvulsive status epilepticus]. Rev Neurol (Paris). 1997;153: 421–426.

95. Liang C-S, Yang F-W. Nonconvulsive status epilepticus in schizophrenia: focus on early detection. J Neuropsychiatry Clin Neurosci. 2011;23: E26. doi:10.1176/jnp.23.4.jnpe26

96. Bottaro FJ, Martinez OA, Pardal MMF, Bruetman JE, Reisin RC. Nonconvulsive status epilepticus in the elderly: a case-control study. Epilepsia. 2007;48: 966–972. doi:10.1111/j.1528-1167.2007.01033.x

97. Labar D, Barrera J, Solomon G, Harden C. Nonconvulsive Status Epilepticus in the Elderly: A Case Series and a Review of the Literature. J Epilepsy. 1998;11: 74–78. doi:10.1016/S0896-6974(97)00134-5

98. Rocco M, Pro S, Alessandri E, Vicenzini E, Mecarelli O. Nonconvulsive status epilepticus induced by acute hypothyroidism in a critically ill patient. Intensive Care Med. 2011;37: 553–554. doi:10.1007/s00134-010-2111-2

99. Fernández-Torre JL, Kaplan PW, Rebollo M, Gutiérrez A, Hernández-Hernández MA, Vázquez-Higuera JL. Ambulatory non-convulsive status epilepticus evolving into a malignant form. Epileptic Disord Int Epilepsy J Videotape. 2012;14: 41–50. doi:10.1684/epd.2012.0488

100. Veran O, Kahane P, Thomas P, Hamelin S, Sabourdy C, Vercueil L. De novo epileptic confusion in the elderly: a 1-year prospective study. Epilepsia. 2010;51: 1030–1035. doi:10.1111/j.1528-1167.2009.02410.x

101. Yilmaz A, Uluc K, Oguz KK, Saygi S. Epileptic nystagmus in a patient with nonconvulsive status epilepticus. Seizure. 2004;13: 183–186. doi:10.1016/S1059-1311(03)00100-6

102. Chang JW, Chang JH, Park SC, Kim TS, Park YG, Chung SS. Radiologically confirmed de novo glioblastoma multiforme and hippocampal sclerosis associated with the first onset of nonconvulsive simple partial status epilepticus. Acta Neurochir (Wien). 2001;143: 297–300; discussion 300-301. doi:10.1007/s007010170110

103. Broderick JP, Cascino TL. Nonconvulsive status epilepticus in a patient with leptomeningeal cancer. Mayo Clin Proc. 1987;62: 835–837. doi:10.1016/s0025-6196(12)62337-4

104. Miranda M, Kuester G, Ríos L, Basaez E, Hazard S. Refractory nonconvulsive status epilepticus responsive to music as an add-on therapy: a second case. Epilepsy Behav EB. 2010;19: 539–540. doi:10.1016/j.yebeh.2010.07.025

105. Narayanan JT, Murthy JMK. Nonconvulsive status epilepticus in a neurological intensive care unit: profile in a developing country. Epilepsia. 2007;48: 900–906. doi:10.1111/j.1528-1167.2007.01099.x

106. Samala RV, Parala-Metz A, Davis MP. Nonconvulsive status epilepticus in a palliative care unit: when delirium is a seizure. Am J Hosp Palliat Care. 2015;32: 243–247. doi:10.1177/1049909113512719

107. Lorenzl S, Mayer S, Noachtar S, Borasio GD. Nonconvulsive status epilepticus in terminally ill patients-a diagnostic and therapeutic challenge. J Pain Symptom Manage. 2008;36: 200–205. doi:10.1016/j.jpainsymman.2007.10.006

108. Hasegawa T, Shiga Y, Narikawa K, Jin K, Fujihara K, Takeda A, et al. Periodic episodes of aphasia as an unusual manifestation of partial status epilepticus. J Clin Neurosci Off J Neurosurg Soc Australas. 2005;12: 820–822. doi:10.1016/j.jocn.2004.09.024

109. Kim T-E, Kim H-J, Park J-H, Lee T-K, Lee JD, Park SA. Cognitive dysfunction with complex visual hallucinations due to focal nonconvulsive status epilepticus: a neuropsychological study and SISCOM. Seizure. 2012;21: 658–660. doi:10.1016/j.seizure.2012.07.001

110. López Arteaga T, Amo C, Serrano González C, Huertas Sánchez D. Nonconvulsive status epilepticus and psychotic symptoms: case report. Riv Psichiatr. 2013;48: 268–270. doi:10.1708/1292.14295

111. Schomer AC, Drislane FW. Severe hemispatial neglect as a manifestation of seizures and nonconvulsive status epilepticus: utility of prolonged EEG monitoring. J Clin Neurophysiol Off Publ Am Electroencephalogr Soc. 2015;32: e4-7. doi:10.1097/WNP.0000000000000107

112. Wakisaka K, Morioka T, Shimogawa T, Murao K, Kanazawa Y, Hagiwara N, et al. Epileptic Ictal Hyperperfusion on Arterial Spin Labeling Perfusion and Diffusion-Weighted Magnetic Resonance Images in Posterior Reversible Encephalopathy Syndrome. J Stroke Cerebrovasc Dis Off J Natl Stroke Assoc. 2016;25: 228–237. doi:10.1016/j.jstrokecerebrovasdis.2015.09.023

113. Ohe Y, Hayashi T, Deguchi I, Fukuoka T, Maruyama H, Kato Y, et al. A case of nonconvulsive status epilepticus with a reversible contralateral cerebellar lesion: temporal changes in magnetic resonance imaging and single-photon emission computed tomography finding. J Stroke Cerebrovasc Dis Off J Natl Stroke Assoc. 2013;22: e639-642. doi:10.1016/j.jstrokecerebrovasdis.2013.03.015

114. Zeiler SR, Turtzo LC, Kaplan PW. SPECT-negative SIRPIDs argues against treatment as seizures. J Clin Neurophysiol Off Publ Am Electroencephalogr Soc. 2011;28: 493–496. doi:10.1097/WNP.0b013e318231c00a

115. Fernández-Torre JL, Agirre Z, Puchades R, Marco De Lucas E, Oterino A. Nonconvulsive status epilepticus causing prolonged stupor after intraventricular hemorrhage: report of a case. Clin EEG Neurosci. 2007;38: 57–60. doi:10.1177/155005940703800112

116. Canas N, Delgado H, Silva V, Pinto AR, Sousa S, Simões R, et al. The electroclinical spectrum, etiologies, treatment and outcome of nonconvulsive status epilepticus in the elderly. Epilepsy Behav EB. 2018;79: 53–57. doi:10.1016/j.yebeh.2017.10.034

117. Chatterjee R, Nagar VS, Sajjan B, Patel K. Nonconvulsive status in the trauma centre: Think of cerebral fat embolism. Neurol India. 2017;65: 1420–1422. doi:10.4103/0028-3886.217950

118. Fernández-Torre JL, Burgueño P, Ballesteros MA, Hernández-Hernández MA, Villagrá-Terán N, de Lucas EM. Super-refractory nonconvulsive status epilepticus secondary to fat embolism: A clinical, electrophysiological, and pathological study. Epilepsy Behav EB. 2015;49: 184–188. doi:10.1016/j.yebeh.2015.04.045

119. Derakhshan I. Nonconvulsive status epilepticus with an unusual EEG: a fresh look at lateralities of motor control and awareness. Epilepsy Behav EB. 2006;9: 204–210. doi:10.1016/j.yebeh.2006.05.005

120. Ibrahim TF, Sweis RT, Nockels RP. Reversible postoperative blindness caused by bilateral status epilepticus amauroticus following thoracolumbar deformity correction: case report. J Neurosurg Spine. 2017;27: 63–67. doi:10.3171/2016.12.SPINE16540

121. Fernández-Torre JL, Paramio-Paz A, Lorda-de Los Ríos I, Martín-García M, Hernández-Hernández MA. Pupillary hippus as clinical manifestation of refractory autonomic nonconvulsive status epilepticus: Pathophysiological implications. Seizure. 2018;63: 102–104. doi:10.1016/j.seizure.2018.11.006

122. Bhatt A, Farooq MU, Bhatt S, Majid A, Kassab MY. Periodic lateralized epileptiform discharges: an initial electrographic pattern in reversible posterior leukoencephalopathy syndrome. Neurol Neurochir Pol. 2008;42: 55–59.

123. Riggio S. Psychiatric manifestations of nonconvulsive status epilepticus. Mt Sinai J Med N Y. 2006;73: 960–966.

124. Warraich S, Ali A, Nizami A, Bakhiet M. Can endotracheal intubation be the first step in management of nonconvulsive status epilepticus?: A case report. Medicine (Baltimore). 2018;97: e9950. doi:10.1097/MD.0000000000009950

125. Blum AS, Drislane FW. Nonconvulsive status epilepticus in thrombotic thrombocytopenic purpura. Neurology. 1996;47: 1079–1081. doi:10.1212/wnl.47.4.1079

126. Garrett WT, Chang CW, Bleck TP. Altered mental status in thrombotic thrombocytopenic purpura is secondary to nonconvulsive status epilepticus. Ann Neurol. 1996;40: 245–246. doi:10.1002/ana.410400218

127. Turtzo LC, Kleinman JT, Llinas RH. Capgras syndrome and unilateral spatial neglect in nonconvulsive status epilepticus. Behav Neurol. 2008;20: 61–64. doi:10.3233/BEN-2008-0210

128. Lim J, Yagnik P, Schraeder P, Wheeler S. Ictal catatonia as a manifestation of nonconvulsive status epilepticus. J Neurol Neurosurg Psychiatry. 1986;49: 833–836. doi:10.1136/jnnp.49.7.833

129. Chicharro-Ciuffardi A, González-Silva M, de Marinis-Palombo A, Gabler-Santalices G. Psychiatric disorders secondary to nonconvulsive status epilepticus of frontal origin. Two clinical case reports. Actas Esp Psiquiatr. 2012;40: 155–160.

130. Kaplan PW, Stagg R. Frontal lobe nonconvulsive status epilepticus: a case of epileptic stuttering, aphemia, and aphasia--not a sign of psychogenic nonepileptic seizures. Epilepsy Behav EB. 2011;21: 191–195. doi:10.1016/j.yebeh.2011.03.028

131. Takaya S, Matsumoto R, Namiki C, Kiyosu H, Isono O, Hashikawa K, et al. Frontal nonconvulsive status epilepticus manifesting somatic hallucinations. J Neurol Sci. 2005;234: 25–29. doi:10.1016/j.jns.2005.02.014

132. Chu K, Kang DW, Kim JY, Chang KH, Lee SK. Diffusion-weighted magnetic resonance imaging in nonconvulsive status epilepticus. Arch Neurol. 2001;58: 993–998. doi:10.1001/archneur.58.6.993

133. Chung PW, Seo DW, Kwon JC, Kim H, Na DL. Nonconvulsive status epilepticus presenting as a subacute progressive aphasia. Seizure. 2002;11: 449–454. doi:10.1053/seiz.2002.0678

134. Flacke S, Wüllner U, Keller E, Hamzei F, Urbach H. Reversible changes in echo planar perfusion- and diffusion-weighted MRI in status epilepticus. Neuroradiology. 2000;42: 92–95. doi:10.1007/s002340050021

135. Shavit L, Grenader T, Galperin I. Nonconvulsive status epilepticus in elderly a possible diagnostic pitfall. Eur J Intern Med. 2012;23: 701–704. doi:10.1016/j.ejim.2012.06.015
